# Predictors of oxidative stress and vascular function in an experimental study of tobacco versus electronic cigarettes: A post hoc analysis of the SUR-VAPES 1 Study

**DOI:** 10.18332/tid/89975

**Published:** 2018-05-08

**Authors:** Simona Mastrangeli, Roberto Carnevale, Elena Cavarretta, Sebastiano Sciarretta, Mariangela Peruzzi, Antonino G. M. Marullo, Elena De Falco, Isotta Chimenti, Valentina Valenti, Christopher Bullen, Leonardo Roever, Giacomo Frati, Giuseppe Biondi-Zoccai

**Affiliations:** 1 Department of Medico-Surgical Sciences and Biotechnologies, Sapienza University of Rome, Latina, Italy; 2 Department of Angiocardioneurology, IRCCS Neuromed, Pozzilli, Italy; 3 Division of Radiology, Sapienza University of Rome, Rome, Italy; 4 National Institute for Health Innovation, School of Population Health, The University of Auckland, Auckland, New Zealand; 5 Department of Clinical Research, Federal University of Uberlândia, Minas Gerais, Brazil

**Keywords:** electronic cigarette, endothelial dysfunction, flow-mediated dilation, oxidative stress, smoking

## Abstract

**INTRODUCTION:**

Use of a conventional cigarette (CC) or electronic cigarette (EC) leads to oxidative stress and endothelial dysfunction, but the impact of other features and their interplay with CCs and ECs have been incompletely appraised. We explored moderators of CC and EC effects on oxidative stress and endothelial dysfunction.

**METHODS:**

We have conducted an experimental study on CCs and ECs in which repeated indicators of oxidative stress (serum levels of soluble NOX2-derived peptide, nitric oxide bioavailability, 8-iso-prostaglandin F2α-III, and vitamin E) and endothelial dysfunction (flow-mediated dilation) were collected in 40 subjects (20 smokers, 20 non-smokers). Several moderating features were appraised, adjusting for smoking status and cigarette type.

**RESULTS:**

Absolute changes in oxidative stress and vascular features after smoking a CC or vaping an EC were significantly correlated (all p<0.05), with the notable exception of 8-iso-prostaglandin F2α-III levels (p=0.030). Inferential analysis based on generalized estimating equations highlighted that the only variable significantly associated with oxidative stress and vascular features was smoking status (all p<0.05). Specifically, we found that smokers had a less pronounced untoward oxidative and vascular response after vaping an EC in comparison to non-smokers, who had oxidative and vascular reactions to an EC that resembled more those seen after smoking a CC. Intriguingly, women taking oral contraceptives appeared to have more unfavorable changes in vitamin E (p=0.002) and FMD (p=0.008).

**CONCLUSIONS:**

This study suggests that the comparative oxidative and vascular effects of an EC versus a CC may be influenced by smoking status, with a potential interaction in women taking oral contraceptives. These findings need further confirmation but could have important clinical and policy implications.

**ABBREVIATIONS:**

SUR-VAPES: Sapienza University of Rome-Vascular Assessment of Proatherosclerotic Effects of Smoking

## INTRODUCTION

Vaping electronic cigarettes (e-cigarettes, hereafter ECs) are an alternative, mistakenly considered safer, nicotine delivery system to conventional cigarettes (CCs), introduced to the commercial market in 2004. Instead of combusting tobacco leaves to produce cigarette smoke, ECs use nicotine or a flavored nicotine-free solution that is heated to produce a vapor. Because no combustion occurs with such devices, ECs are widely used as cigarette substitutes for reducing cigarette consumption or quitting tobacco altogether. ECs include a wide range of devices operating with batteries and using different aerosol delivery methods. Electronic cigarette liquids are usually made of a mixture of glycerol and propylene glycol, flavors, and optionally variable concentrations of nicotine ranging from 1.6 to 19 mg per cartridge, with more variable concentrations for tank systems^1^.

However, benefits and risks of ECs remain the subject of debate among policy makers and researchers^2-4^. Indeed, only relatively few studies on the biological and health effects of ECs have been performed, and mainly focusing on their chemical composition and/or estimation of their toxicological exposure^5^, reporting preliminary evidence of subclinical toxicity, including untoward effects on pulmonary function, vascular function and oxidative stress. In particular, Carnevale et al.^1^ have shown that both an EC or a CC may have unfavorable effects on markers of oxidative stress and flow-mediated dilation (FMD) even after single use, although ECs seem to have a lesser impact. It remains unclear, though, whether individual subject features may impact on biologic responses to different types of smoking.

We thus aimed to identify individual predictors of detrimental effects of ECs and CCs on oxidative stress and endothelial dysfunction in a cohort of healthy subjects without cardiovascular disease.

## METHODS

### Design

This is a post hoc analysis of a prospective study that has been reported in detail elsewhere^1^. Briefly, the SUR-VAPES 1 trial was a cross-over single-blind trial, conducted from September 2014 to March 2015 at Sapienza University of Rome, and included 20 healthy smokers and 20 healthy non-smokers, matched for age and gender. All participants gave written informed consent, and the trial was approved by Sapienza University of Rome ethical committee (06-27-2014, protocol number 813/14) and was conducted in accordance with the principles of the Declaration of Helsinki.

### Subjects

Subjects were defined as healthy on the basis of the following: 1) no history of acute or chronic organic, metabolic, and inflammatory diseases; 2) no fever or infections in the last 3 months; 3) no history of cardiovascular pathologic symptoms; 4) no allergies; and 5) normal blood pressure levels and heart rhythm. Women were not menstruating when the experiment was performed. Notably, no subject reported diabetes mellitus, and in the month preceding and during the study, none of the participants took vitamin E, other antioxidant supplements, or other drugs that could potentially affect oxidative stress or FMD.

### Procedures

After a washout of 1 month in case of smoking history, 40 subjects (20 smokers, 20 non-smokers) underwent blood draws for measurement of biomarkers, additional blood tests and brachial FMD. Smoking history (time of initiation) and intensity (cigarettes per day) were self-reported, but abstinence was confirmed with a blood cotinine test administered before each experimental smoking session. Specifically, liquid chromatography/tandem mass spectrometry was employed (Quest Diagnostics) with a 3 ng/mL cut-off.

They thus were instructed to use either an EC (charged with a nicotine cartridge, with a mean nicotine content of 16 mg, equivalent to 250 puffs, with subjects taking nine puffs, approximately 0.6 mg of nicotine) or a CC (with a mean nicotine content of 0.6 mg). Immediately after smoking, the above measurements were repeated. Subsequently, after an additional wash-out of one week, with abstinence again confirmed with a formal cotinine assay, the same procedure was followed but using the other product to enable within-subject comparisons.

### Endpoints

The endpoints of the study were markers of oxidative balance/stress and endothelial dysfunction, which have important pathophysiologic and prognostic roles in atherothrombosis. Thus, through an acute in vivo study^6,7^, precise and informative insights were provided based on: 1) serum levels of soluble NOX2-derived peptide (sNOX2-dp), 2) serum levels of nitric oxide (NO) bioavailability, 3) serum levels of 8-iso-prostaglandin F2α-III (8-iso-PGF2α-III), 4) serum levels of vitamin E, and 5) brachial FMD.

Specifically, sNOX-2-dp was measured using an enzyme-linked immunosorbent assay as described by Loffredo et al^8^. Nitric oxide bioavailability was measured with a colorimetric assay kit (Abcam, DRG International), 8-iso-PGF2α-III was appraised with a colorimetric assay kit (Abcam, DRG International), and vitamin E was analyzed using an Agilent 1200 Infinity series high-performance liquid chromatography system (Agilent Technologies, equipped with an Eclipse Plus C18 column), with results presented as the ratio between the concentration of a-tocopherol and serum total cholesterol. Brachial FMD was measured with a standardized and validated procedure8.

### Analysis

For descriptive purposes, we computed differences between baseline and post-exposure levels for each endpoint of interest, dividing them into tertiles, thus creating three groups for each endpoint. We then reported for each tertile the corresponding continuous variables as mean (standard deviation) and the categorical variables as count (percentage). Groups derived by tertiles for each endpoint were then compared with analysis of variance for continuous variables and Fisher exact test for categorical variables. The main inferential analysis was however based on generalized estimating equation methods, with an unstructured variance-covariance specification, forcing in the model each biomarker and FMD value, timing of sampling, cigarette type, smoking status, and one of the following clinical features, iteratively: age, gender, height, weight, body surface area, body mass index, systolic blood pressure, diastolic blood pressure, cholesterol, time since smoking initiation , cigarettes per day, and drug therapy. Such analyses were reported as point estimates of effect (95% confidence intervals), and corresponding p-values. In addition, exploratory linear correlation and linear regression were used to explore the association between changes in the different biomarker and FMD values. Statistical significance was set at the 2-tailed 0.05 level, without a multiplicity adjustment. Computations were performed with R (R Foundation for Statistical Computing, Vienna, Austria) and Stata 13 (StataCorp, College Station, TX, USA).

## RESULTS

Absolute changes in oxidative stress and vascular features after smoking a CC and vaping an EC were significantly associated (all p<0.05), with the notable exception of 8-iso-prostaglandin F2α-III levels (p=0.030; Supplementary Table 1S; Supplementary Figures 1S to 5S).

Descriptive bivariate analysis encompassing all 40 included subjects is detailed in Tables 1 to 4. Specifically, there were no apparently significant differences in baseline features after stratification of participants in tertiles of absolute differences in serum levels of sNOX2-dp. Conversely, increasing height appeared associated with more favorable changes in NO (173.6±9.1 cm in the first tertile vs 168.5±11.4 cm in the second tertile vs 167±8.7 cm in the third tertile, p=0.015), as was diastolic blood pressure (77.9±8.4 mm Hg vs 71.7±11.4 mm Hg vs 72.5±7.9mm Hg, p=0.039). Analysis of baseline vs post-exposure levels in 8-iso-PGF2α-III showed a significant association between prior smoking burden (measured as years since smoking initiation) and 8-iso-PGF2α-III production (3.8±3.9 years vs 4.2±4.5 years vs 1.5±2.7 years, p=0.031). No predictors of changes in vitamin E and FMD (Table 5) were instead identified.

**Table 1:** Descriptive analysis based on tertiles of differences between baseline and post-exposure levels of soluble NOX2-derived peptide*

*Feature*	*1st tertile (N=25)*	*2nd tertile (N=28)*	*3rd tertile (N=26)*	*p*
Age (years)	27.9±4.7	28.6±5.6	27.3±5.5	0.676
Female gender	11 (44.0%)	17 (60.7%)	14 (51.9)	0.480
Height (cm)	169.3±9.3	169.6±10.0	170.2±11.1	0.758
Weight (kg)	67.3±11.2	67.9±12.2	66.6±14.9	0.846
Body surface area (m^2^)	1.8±0.2	1.8±0.2	1.8±0.2	0.892
Body mass index (kg/cm^2^)	23.4±2.6	23.5±2.4	22.8±3.5	0.455
Systolic pressure (mm Hg)	122.7±20.1	116.5±10.3	118.0±8.7	0.227
Diastolic pressure (mg Hg)	74.9±9.8	73.3±11.2	74.0±7.7	0.752
Cholesterol (mg/dL)	179.7±13.7	181.0±15.0	179.5±11.0	0.958
Smoking status	11 (44%)	15 (53.6%)	14 (51.9%)	0.787
Smoking since	2.6±3.2	3.5±4.1	3.4±4.3	0.450
Cigarettes per day	5.5±7.8	7.2±8.3	6.9±7.1	0.534
Drug therapy				
Fluvoxamine	0 (0%)	3 (10.7%)	1 (3.7%)	0.318
Lavitirantam	2 (8%)	0 (0%)	0 (0%)	0.095
Oral contraceptive	2 (8.0%)	2 (7.1%)	2 (7.4%)	1

* Each patient provided two differences; the last group corresponds to the one with higher soluble NOX2-derived peptide generation; Analysis was based on analysis of variance for continuous variables and Fisher exact test for categorical variables.

**Table 2:** Descriptive analysis based on tertiles of differences between baseline and post-exposure levels in nitric oxide production*

*Feature*	*1st tertile (N=27)*	*2nd tertile (N=26)*	*3rd tertile (N=27)*	*p*
Age (years)	26.8±4.0	28.2±5.5	28.9±6.0	0.148
Female gender	10 (37%)	16 (61.5%)	16 (59.3%)	0.152
Height (cm)	173.6±9.1	168.5±11.4	167±8.7	0.015
Weight (kg)	70.9±10.8	65.4±14.3	65.5±12.6	0.121
Body surface area (m^2^)	1.8±0.2	1.7±0.2	1.7±0.2	0.065
				
Body mass index (kg/cm^2^)	23.5±3.2	22.8±2.8	23.3±2.6	0.746
				
Systolic pressure (mm Hg)	118.1±7.2	120.8±16.8	118.0±15.8	0.961
				
Diastolic pressure (mg Hg)	77.9±8.4	71.7±11.4	72.5±7.9	0.039
				
Cholesterol (mg/dL)	182.9±12.7	177.5±15.9	179.7±10.34	0.372
Smoking status	14 (51.9%)	11 (42.3%)	15 (55.6%)	0.666
Smoking since	2.6±2.6	3.1±4.6	3.9±4.3	0.243
Cigarettes per day	6.4±6.8	5.3±6.5	7.9±9.6	0.507
Drug therapy				
Fluvoxamine	1 (3.7%)	3 (11.5%)	0 (0%)	0.120
Lavitirantam	1 (3.7%) 0 (0%)		1 (3.7%)	1
Oral contraceptive	2 (7.4%)	2 (7.7%)	2 (7.4%)	1

* Each patient provided two differences; the last group corresponds to the one with higher nitric oxide consumption.

**Table 3:** Descriptive analysis based on tertiles of differences between baseline and post-exposure levels in 8-iso-prostaglandinF2α-III*

*Feature*	*1st tertile (N=27)*	*2nd tertile (N=26)*	*3rd tertile (N=27)*	*p*
Age (years)	27.4±5.7	28.9±4.8	27.6±5.4	0.898
Female gender	13 (48.1%)	15 (57.7%)	14 (51.9%)	0.817
Height (cm)	170.9±10.6	169.2±9.5	169.0±10.4	0.503
Weight (kg)	67.1±13.2	67.8±13.9	66.9±11.4	0.966
Body surface area (m^2^)	1.8±0.2	1.8±0.2	1.8±0.2	0.874
Body mass index (kg/cm^2^)	22.8±3.2	23.5±3.2	23.3±2.1	0.567
Systolic pressure (mg Hg)	120.6±16.2	115.8±8.7	120.4±15.2	0.969
Diastolic pressure (mm Hg)	74.1±7.6	75.5±10.9	72.6±10.2	0.565
Cholesterol (mg/dL)	178.0±11.2	185.3±9.7	177.0±16.4	0.782
Smoking status	16 (59.3%)	16 (61.5%)	8 (29.6%)	**0.043**
Smoking since	3.8±3.9	4.2±4.5	1.5±2.7	**0.031**
Cigarettes per day	7.9±8.3	7.5±6.9	4.3±7.7	0.081
Drug therapy				
Fluvoxamine	2 (7.4%)	2 (7.7%)	0 (0%)	0.4607
Lavitirantam	1 (3.7%)	1 (3.8 %)	0 (0%)	0.769
Oral contraceptive	1 (3.7%)	2 (7.7%)	3 (11.1%)	0.689

* Each patient provided two differences; the last group corresponds to the one with higher 8-iso-prostaglandin F2a generation.

**Table 4:** Descriptive analysis based on tertiles of differences between baseline and post-exposure levels in vitamin E*

*Feature*	*1st tertile (N=26)*	*2nd tertile (N=27)*	*3rd tertile (N=26)*	*p*
Age (years)	28.6±5.3	28.3±5.1	27.0±5.4	0.246
Female gender	12 (44.4%)	17 (65.4%)	13 (48.1%)	0.283
Height (cm)	171.1±10.3	167.4±9.2	170.5±10.6	0.841
Weight (kg)	68.7±12.7	66.6±13.6	66.5±13.6	0.539
Body surface area (m^2^)	1.8±0.2	1.8±0.2	1.8±0.2	0.588
Body mass index (kg/cm^2^)	23.3±2.4	23.6±3.4	22.7±2.7	0.489
Systolic pressure (mg Hg)	121.4±20.2	116.7±8.6	118.7±9.6	0.476
Diastolic pressure (mm Hg)	73.7±9.6	74.7±10.2	73.9±9.4	0.944
Cholesterol (mg/dL)	177.1±14.2	183.5±13.2	179.7±11.7	0.467
Smoking status	15 (55.6%)	15 (57.7%)	10 (37%)	**0.295**
Smoking since	3.0±3.7	4.0±4.3	2.5±3.8	**0.608**
Cigarettes per day	7.5±7.9	6.0±6.3	6.1±9.0	0.518
Drug therapy				
Fluvoxamine	0 (0%)	3 (11.5%)	1 (3.7%)	0.120
Lavitirantam	1 (3.7%)	1 (3.8%)	0 (0%)	0.769
Oral contraceptive	4 (14.8%)	0 (0%)	2 (7.4%)	0.157

* Each patient provided two differences; the last group corresponds to the one with higher vitamin E consumption.

**Table 5:** Descriptive analysis based on tertiles of differences between baseline and post-exposure levels in flow-mediated dilation (FMD)*

*Feature*	*1st tertile (N=27)*	*2nd tertile (N=26)*	*3rd tertile (N=27)*	*p*
Age (years)	28.4±6.2	29.3±4.3	26.5±5.1	0.259
Female gender	17 (63%)	14 (53.8%)	7 (31.8%)	0.085
Height (cm)	170.2±11.2	169.0±8.8	171.0±10.4	0.799
Weight (kg)	66.1±11.6	67.2±12.2	70.9±14.4	0.195
Body surface area (m^2^)	1.8±0.2	1.8±0.2	1.8±0.2	0.292
Body mass index (kg/cm^2^)	22.7±2.3	23.4±3.2	24.0±2.9	0.120
Systolic pressure (mg Hg)	119.4±16.1	121.5±15.9	117.4±7.8	0.668
Diastolic pressure (mm Hg)	72.0±9.5	75.2±10.0	76.1±9.1	0.122
Cholesterol (mg/dL)	179.0±15.2	181.0±9.7	179.6±14.9	0.845
Smoking status	12 (44.4%)	17 (65.4%)	8 (36.4%)	0.118
Smoking since	2.7±3.2	4.9±4.8	1.6±2.6	0.432
Cigarettes per day	5.2±6.3	8.8±8.5	5.2±8.2	0.909
Drug therapy				
Fluvoxamine	1 (3.7%)	3 (11.5%)	0 (0%)	0.258
Lavitirantam	0 (0%)	0 (0%)	2 (9.1%)	0.083
Oral contraceptive	2 (7.4%)	4 (15.4%)	0 (0%)	0.200

* Each patient provided two differences; the last group corresponds to the one with lower FMD.

Inferential analysis, based on generalized estimating equations, highlighted that the only variable strongly, uniformly and significantly associated with oxidative stress and vascular features was smoking status (all p<0.05; Table 6; Supplementary Figure 1S to 5S). Specifically, we found that smokers had a less pronounced untoward oxidative and vascular response after vaping an EC in comparison to non-smokers, who had oxidative and vascular reactions to an EC that resembled more those seen after smoking a CC.

**Table 6:** Inferential analysis based on generalized estimating equations to identify independent predictors in oxidative stress and vascular function parameters*

*Feature*	*Soluble NOX2-derived peptide*	*Nitric oxide production*	*8-iso-prostaglandin F2a*	*Vitamin E*	*Flow-mediated dilation*
Age	0.070 (-1.47; 0.26) p=0.528	-0.04 (-0.30; 0.22) p=0.761	0.098 (-1.12; 1.318) p=0.875	0.01 (-0.05; 0.06) p=0.731	0.09 (-0.45; 0.23) p=0.192
Female gender	0.33 (-2.59; 1.92) p=0.772	-0.27 (-2.96; 2.41) p=0.842	2.27 (-10.36; 14.90) p=0.725	0.23 (-0.33; 0.79) p=0.426	0.61 (-0.85; 2.07) p=0.413
Height	-0.03 (-0.14; 0.09) p=0.622	-0.03 (-0.14; 0.07) p=0.330	0.27 (-0.38; 0.91) p=0.417	0.00 (-0.03; 0.03) p=0.907	-0.03 (-0.10; 0.05) p=0.487
Weight	0.02 (-0.68; 0.11) p=0.639	-0.07 (-0.20; 0.07) p=0.540	0.24 (-0.25; 0.74) p=0.340	0.01 (-0.02; 0.03) p=0.530	-0.01 (-0.07; 0.05) p=0.800
Body surface area	0.76 (-4.61; 6.13) p=0.780	-2.48 (-8.84; 3.88) p=0.445	14.59 (-15.25; 44.43) p=0.338	0.31 (-1.03; 1.66) p=0.647	0.63 (-4.13; 2.87) p=0.723
Body mass index	0.28 (-0.11; 0.67) p=0.155	-0.06 (-0.53; 0.42) p=0.816	0.66 (-1.57; 2.88) p=0.562	0.03 (-0.07; 0.13) p=0.517	0.07 (-0.19; 0.33) p=0.610
Systolic pressure	0.01 (-0.07; 0.09) p=0.816	-0.06 (-0.15; 0.04) p=0.589	0.16 (-0.29; 0.62) p=0.481	-0.01 (-0.03; 0.02) p=0.626	0.02 (-0.04; 0.07) p=0.562
Diastolic pressure	0.02 (-0.10; 0.14) p=0.764	-0.04 (-0.18;0.10) p=0.589	-0.07 (-0.73; 0.60) p=0.836	0.01 (-0.02; .039) p=0.545	0.00 (-0.08; 0.08) p=0.985
Cholesterol	0.03 (-0.06; 0.12) p=0.497	0.01 (-0.09; 0.11) p=0.854	-0.14 (-0.63; 0.34) p=0.558	0.00 (-0.02; 0.03) p=0.694	0.00 (-0.06; 0.06) p=0.975
Smoking history	**9.29 (5.88; 12.70) p<0.001**	**-17.34 (-21.10; -13.57) p<0.001**	**109.54 (93.05; 126.02) p<0.001**	**-0.99 (-1.85; -0.13) p=0.021**	**-2.20 (-4.15; -0.26) p=0.020**
Smoking since	0.20 (-0.29; 0.68) p=0.428	0.02 (-0.57; 0.60) p=0.955	0.70 (-2.04; 3.44) p=0.618	-0.07 (-0.19; 0.05) p=0.247	0.09 (-0.23; 0.41) p=0.575
Cigarettes per day	-0.11 (-0.39; 0.17) p=0.431	0.15 (-0.18; 0.48) p=0.382	0.35 (-1.22; 1.93) p=0.661	-0.05 (-0.11; 0.02) p=0.198	-0.01 (-0.19; 0.18) p=0.952
Drug therapy					
Fluvoxamine	2.64 -2.60; 7.88) p=0.323	-4.86 (-11.00; 1.27) p=0.120	7.18 (-22.49; 6.85) p=0.635	-0.47 (-1.79; 0.85) p=0.487	1.34 (-2.10; 4.77) p=0.446
Levatiracetam	0.20 (-0.29; 0.68) p=0.958	-6.67 (-15.11; 1.77) p=0.121	**44.95 (6.44; 83.46) p=0.022**	-0.34 (-2.16; 1.49) p=0.719	-0.05 (-4.80; 4.72) p=0.984
Oral contraceptive	-2.18 (-6.42; 2.05) p=0.313	-2.79 (-7.83; 2.24) p=0.277	-1.10 (-25.16; 22.97) p=0.929	**1.55 (0.59; 2.51) p=0.002**	**3.47 (0.89; 6.05) p=0.008**

* Adjusted for smoking status and cigarette type, and reported as point estimates of effect (95% confidence intervals), and corresponding p values.

Inferential analysis also suggested that the use of an oral contraceptive pill and levetiracetam consumption could be significant predictors of vitamin E and FMD, on the one hand, and of 8-iso-PGF2α-III, on the other (all p<0.05; Supplementary Figure 6S to 8S). While the latter association is likely due to small sample effects (Supplementary Figure 6S), women using the oral contraceptive pill appeared to have more unfavorable changes in vitamin E (p=0.002) and FMD (p=0.008).

## DISCUSSION

This post hoc analysis of the SUR-VAPES 1 trial suggests that the comparative oxidative and vascular effects of EC versus CC may be influenced by smoking status, with a potential interaction due to oral contraceptives. These findings need further confirmation but could have important clinical and policy implications.

Smoking is a significant independent risk factor for chronic obstructive pulmonary disease (COPD) and cardiovascular disease, in particular, coronary artery and cerebro-vascular diseases. The leading cause of structural and functional alterations to the cardiovascular and respiratory systems seems to be related to oxidative stress, endothelial dysfunction and persisting inflammation^9,10^. The number of cigarettes smoked plays an important role in increasing the level of oxidative damage and reducing antioxidant defense, important for coronary artery disease (CAD),^11^. Given the craving of smokers and the quest for safer alternatives, novel devices to deliver tobacco products have been proposed, with the ultimate goal of reducing morbidity and mortality while maintaining palatability. Electronic cigarettes represent thus an emerging topic of interest for clinicians and researchers. Whilst ECs may appear at first glance safer than CCs, emerging evidence suggest that they can also have untoward effects. Most recently, the use of ECs has been seen as a potential strategy to increase cessation rates of CCs^12^.

In our study, we find that a more favorable change in NO was associated with increasing height and with a decreasing diastolic blood pressure, probably associated with a better hemodynamic condition. Higher NO serum level appeared to be related to less oxidative stress and more vasodilatation^13^. Analysis of baseline vs post-exposure levels in 8-iso-PGF2α-III suggests a significant association between prior smoking burden and 8-iso-PGF2α-III production. Increasing 8-iso-PGF2α-III production is associated with a higher oxidative stress and with acute myocardial infarction^14^. These data were however not confirmed by multivariable analysis.

Our study thus suggests that smoking status may be significantly associated with oxidative stress and vascular changes. Specifically, we found that smokers appeared to have a less pronounced untoward oxidative and vascular response after vaping an EC in comparison to non-smokers, who had oxidative and vascular reactions to an EC that were similar to those seen after smoking a CC. That could be partially explained by the pre-conditioning effect of cigarettes on the subject that is not present in non-smokers. That could have important implications; for example, if our findings are confirmed, then they would reinforce the recommendation that EC should be used by CC users as a safer product but not by non-smokers for recreational purposes. Previously, Moheimani et al.^15^ have confirmed that ECs are associated with oxidative stress (appraised by low-density lipoprotein oxidizability), in agreement with our main findings. Most recently, the same authors have reported a randomized trial comparing nicotine-containing EC, non-nicotine-containing EC, and sham devices, focusing on heart rate variability and oxidative stress (appraised by plasma paraoxonase activity). Their results suggest that most biologic effects of ECs are the result of nicotine exposure only.

Furthermore, our study suggests that oral contraceptives could interplay with vitamin E, FMD and 8-iso-PGF2α-III; women using oral contraceptives appear to have more unfavorable changes in vitamin E, FMD and of 8-iso-PGF2α-III after vaping an EC. This finding needs confirmation by other studies, as its implications are important, especially in light of the possibility of additional untoward interactions with flavorings and additives commonly used in ECs, and their long-term use. Previously, Halley et al. found rare cardiovascular outcomes among EC users using contraceptives, suggesting that more research is needed on the potential detrimental interaction between oral contraceptives, EC exposure and clinical outcomes^16^.

### Limitations

Limitations of the present study include: the lack of randomization, which might have led to selection bias; the appraisal only of the acute effects of CC and EC exposures, impeding the generalization to chronic use of these tobacco products; the selection of healthy volunteers, which limits the external validity of our findings towards patients with established cardiovascular disease; and the focus only on surrogate endpoints, further limiting implications for clinical practice. Furthermore, we did not test subjects with a sham product, and thus confounding effects due to awareness of the type of smoking and timing of sampling cannot be excluded. Most importantly, we did not adjust for multiple testing, thus inflating the risk of Type I error. Accordingly, future studies may confirm or disprove the present findings. In addition, such studies could investigate arterial stiffness, focusing on the chronic effect of EC vs CC use. Additionally, it would be interesting to analyze the effect on oxidative stress and endothelial function of EC without nicotine, adding a non-vapor EC, a heat-not-smoke cigarette, or a sham device.

## CONCLUSIONS

This study provides preliminary evidence that the comparative oxidative and vascular effects of EC and CC may be influenced by smoking status, with a potential interaction due to oral contraceptive use. These findings need further confirmation, as they could have important clinical and policy implications.

## CONFLICTS OF INTEREST

G. Biondi-Zoccai reports personal fees from Abbott Vascular, and personal fees from Bayer, outside the submitted work. The rest of the authors have also completed and submitted an ICMJE form for disclosure of potential conflicts of interest. The authors declare that they have no competing interests, financial or otherwise, related to the current work.
