# Supplementary material for: Predictors of oxidative stress and vascular function in an experimental study of tobacco versus electronic cigarettes: A post hoc analysis of the SUR-VAPES 1 Study
Source: Tob Induc Dis. 2018 May 8;16:18. doi: 10.18332/tid/89975 (PMC6659516; doi:10.18332/tid/89975)

Online supplement to:

Predictors of oxidative stress and vascular function in an experimental study of tobacco versus electronic cigarettes: A post hoc analysis of the SUR-VAPES 1 Study

Table 1S. *Association between baseline and post-exposure changes in oxidative and vascular features after smoking a combustion cigarette and an electronic cigarette*

**Variable**	**R (95% confidence interval)**	**p**
Soluble NOX2-derived peptide	0.333 (0.024; 0.584)	0.036
Nitric oxide production	0.643 (0.414; 0.800)	<0.001
8-iso-prostaglandin F2a	0.168 (-0.162; 0.447)	0.330
Vitamin E	0.462 (0.175; 0.676)	0.003
Flow-mediated dilation	0.309 (-0.003;0.566)	0.053

Figure 1S. Association between baseline and post-exposure changes in soluble NOX2-derived peptide after smoking a combustion cigarette and an electronic cigarette (p=0.036). Positive values indicate increased oxidative stress. Red dots indicate smokers, blue triangles indicate non-smokers

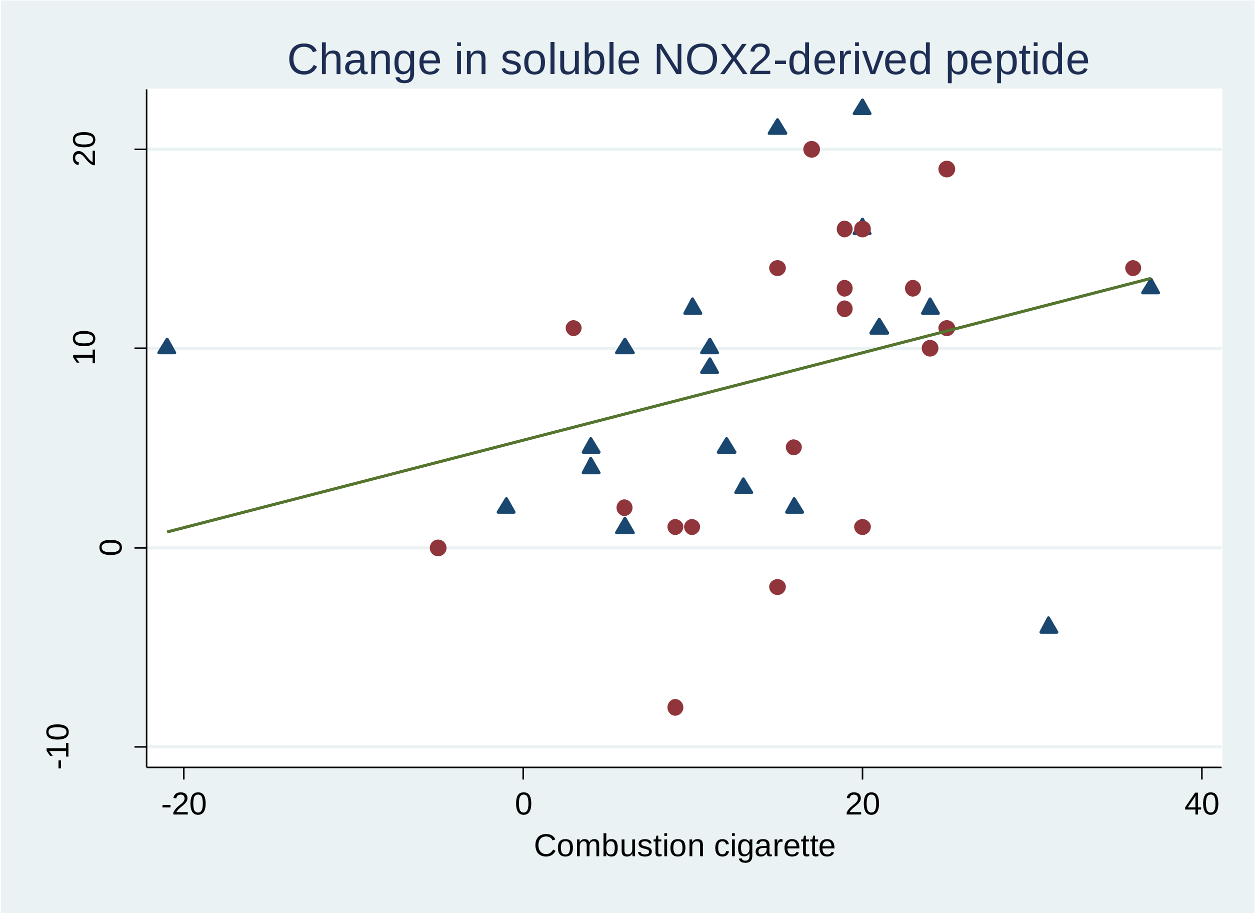

Figure 2S. Association between baseline and post-exposure changes in nitric oxide (NO) production after smoking a combustion cigarette and an electronic cigarette (p<0.001). Negative values indicate increased oxidative stress. Red dots indicate smokers, blue triangles indicate non-smokers

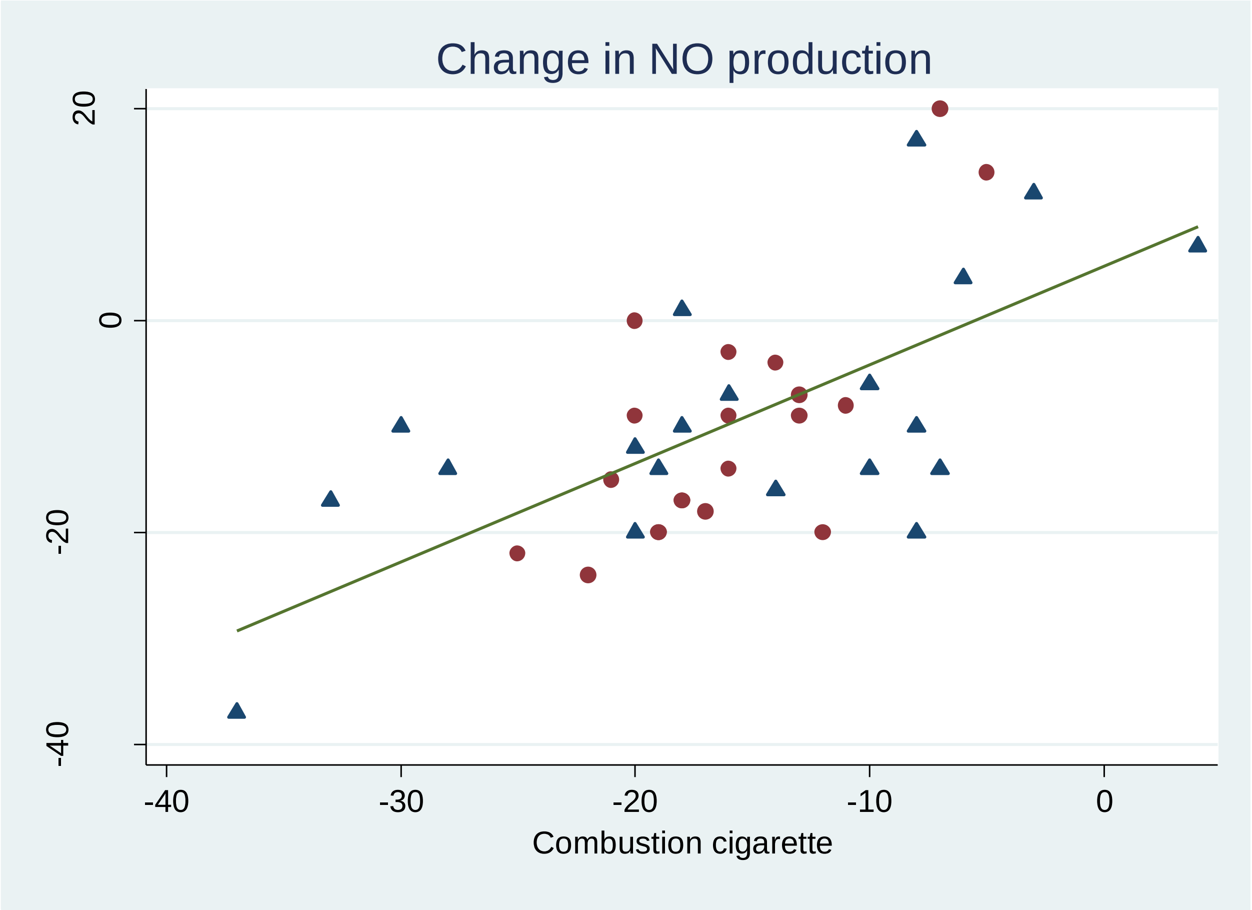

Figure 3S. Association between baseline and post-exposure changes in levels of in 8-iso-prostaglandin F2α-III after smoking a combustion cigarette and an electronic cigarette (p=0.330). Positive values indicate increased oxidative stress. Red dots indicate smokers, blue triangles indicate non-smokers

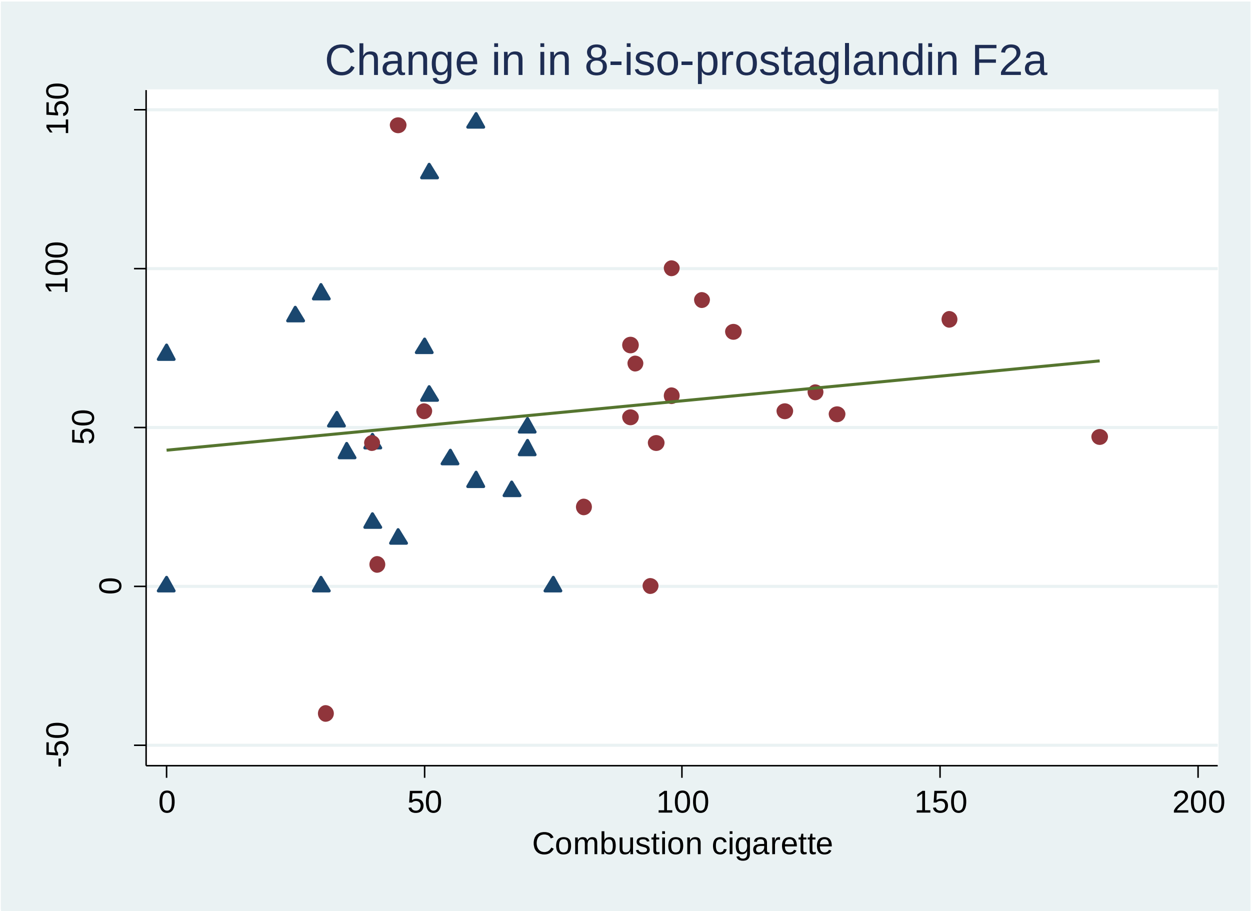

Figure 4S. Association between baseline and post-exposure changes in levels of vitamin E after smoking a combustion cigarette and an electronic cigarette (p=0.003). Negative values indicate increased oxidative stress. Red dots indicate smokers, blue triangles indicate non-smokers

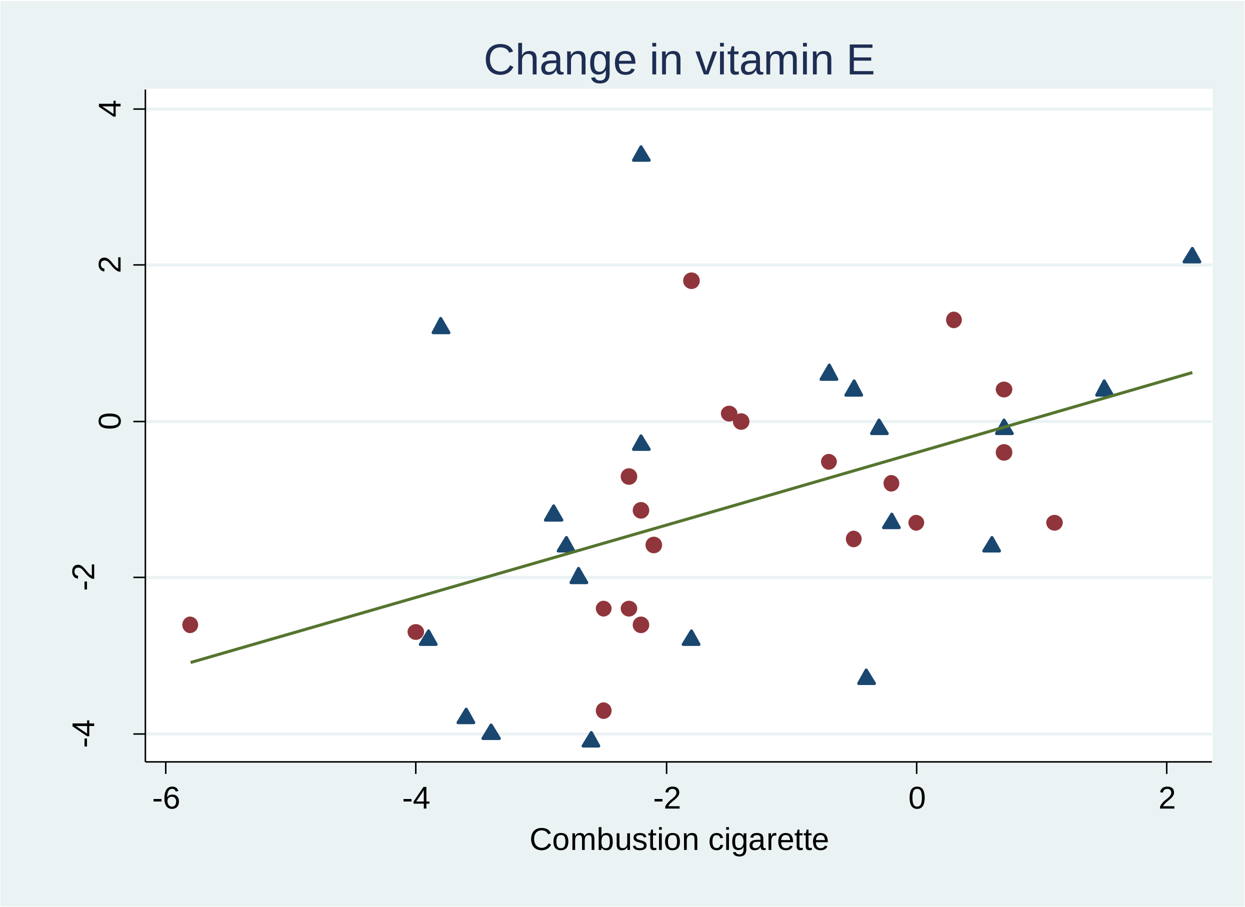

Figure 5S. Association between baseline and post-exposure changes in flow-mediated dilation (FMD) after smoking a combustion cigarette and an electronic cigarette (p=0.053). Negative values indicate worse vascular function. Red dots indicate smokers, blue triangles indicate non-smokers

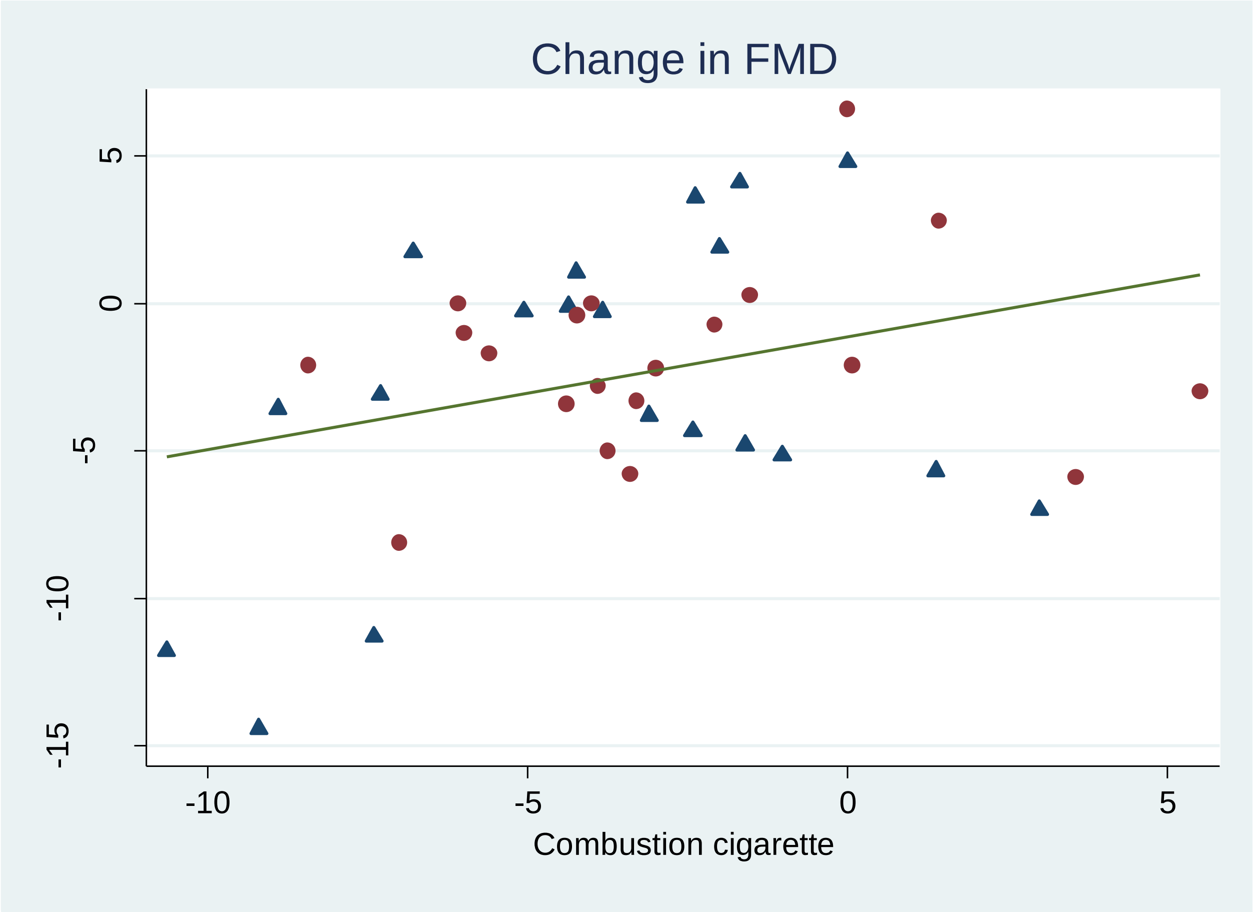

Figure 6S. Association between baseline and post-exposure changes in levels of in 8-iso-prostaglandin F2α-III after smoking a combustion cigarette and an electronic cigarette (p=0.330). Positive values indicate increased oxidative stress. Red dot indicates the subject using levetiracetam, blue triangles indicate the other subjects

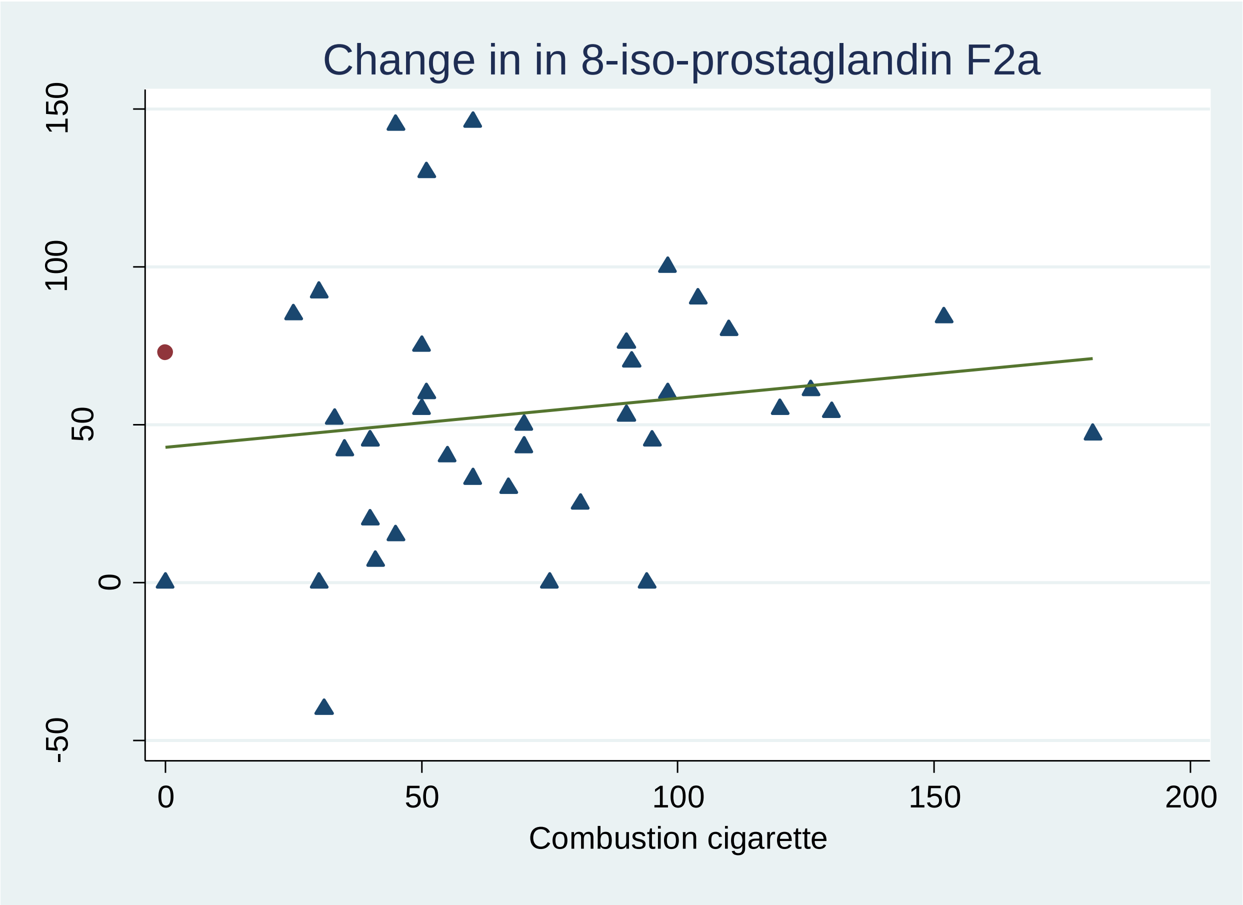

Figure 7S. Association between baseline and post-exposure changes in levels of vitamin E after smoking a combustion cigarette and an electronic cigarette (p=0.003). Negative values indicate increased oxidative stress. Red dots indicate subjects using oral contraceptives, blue triangles indicate the others

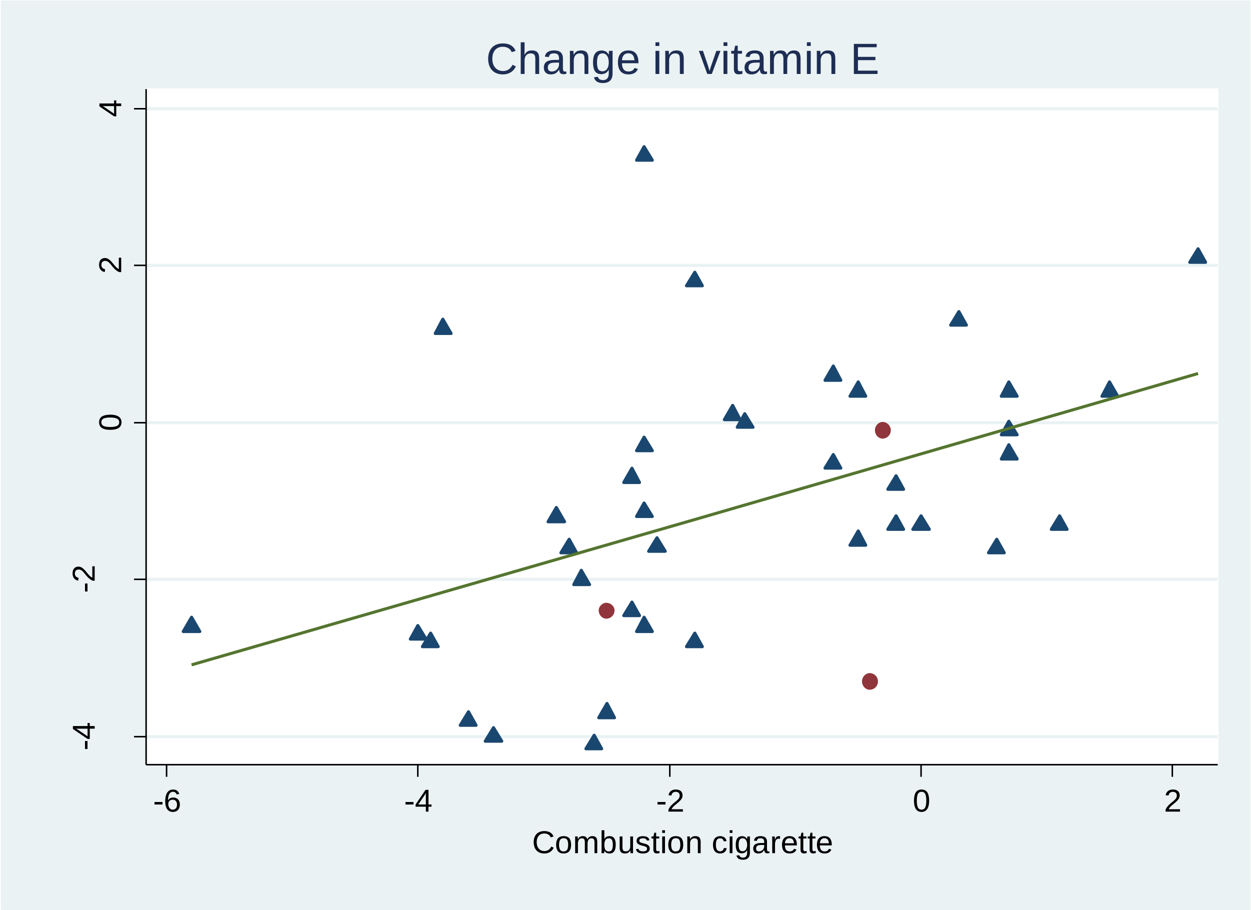

Figure 8S. Association between baseline and post-exposure changes in flow-mediated dilation (FMD) after smoking a combustion cigarette and an electronic cigarette (p=0.053). Negative values indicate worse vascular function. Red dots indicate subjects using oral contraceptives, blue triangles indicate the others

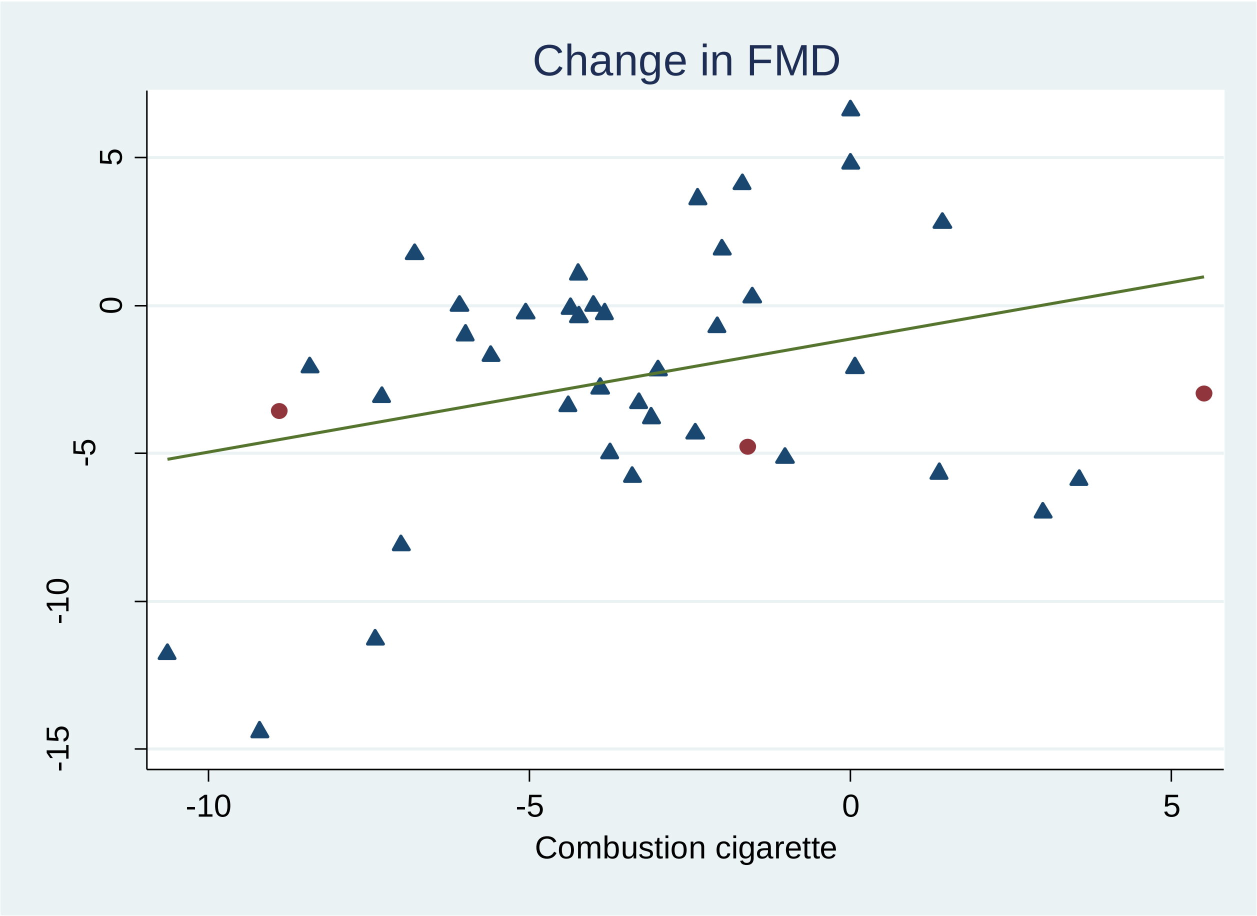

Supplement: Supplementary file 1 [file TID-16-18-s1.pdf]
